# A Sixteenth-Century Complaint

**Published:** 1912-07-06

**Authors:** 


### A SIXTEENTH-CENTURY COMPLAINT.

Of "Gammer Gurton's Needle," perhaps the earliest comedy in the language, and notable also for containing a version of the old drinking song, "Back and Side, go bare," the argument is as follows: The Gammer is mending a rent in her man Hodge's nether garment, when, her attention being distracted suddenly, she loses her needle. In those days this loss was a serious one, and the whole household falls to searching for the needle, but unavailingly. At last a friend who is fond of practical joking persuades her that her neighbour, Dame Chat, has stolen it. The two old women come to high words, and, worse, to blows, about the matter. Still the needle is not found, and in her trouble Gammer Gurton decides to take advice of Doctor Rat. Doctor Rat is not a doctor of medicine, but the curate. Nevertheless, he seems to have medical dealings with his flock, and his remarks on the summons, bating the mediæval vigour of the language, are so like what a twentieth-century colliery surgeon might say in his haste, that it may be of interest to quote them:—

"A man were better twenty times be a bandog and bark,
Than here among such a sort be parish priest or clerk,
Where he shall never be at rest one pissing while a day,
But he must trudge about the town, this way and that way;
Here to a drab, there to a thief, his shoes to tear and rent,
And that which is worst of all, at every knave's commandment!
I had not sit the space to drink two pots of ale,
But Gammer Gurton's sorry boy was straightway at my tail,

And she was sick, and I must come, to do I wot not what!
If once her finger's end but ache—trudge, call for Doctor Rat!
And when I come not at their call, I only thereby lose;
For I am sure to lack therefore a tithe-pig or a goose.

I warrant you, when truth is known, and told they have their tale,
The matter whereabout I come is not worth a halfpenny-worth of ale;
Yet must I talk so sage and smooth, as though I were a gloser,
Else ere the year come at an end I shall be sure the loser."

The present call proves an especially unfortunate one for poor Dr. Rat, as Diccon, the joker aforesaid, contrives to get plenty of fun out of him too. Ultimately Diccon smartly transfers the needle to the luckless Hodge, who, innocently withdrawing it from a part of his person possessing marked anatomical advantages, gives it to his mistress and all the trouble is at an end.

"'Tis mine own dear nee'le, Hodge, sikerly I wot!"

